# Application of quantitative trait locus mapping and transcriptomics to studies of the senescence-accelerated phenotype in rats

**DOI:** 10.1186/1471-2164-15-S12-S3

**Published:** 2014-12-19

**Authors:** Elena E Korbolina, Nikita I Ershov, Leonid O Bryzgalov, Natalia G Kolosova

**Affiliations:** 1 Institute of Cytology and Genetics, SB RAS, Novosibirsk, Russia; 2 Novosibirsk State University, Novosibirsk, Russia

**Keywords:** Senescence-accelerated OXYS rats, Age-related macular degeneration, Quantitative trait locus, Congenic strain, RNA-Seq, Non-synonymous SNP, Retinal transcriptome

## Abstract

**Background:**

Etiology of complex disorders, such as cataract and neurodegenerative diseases including age-related macular degeneration (AMD), remains poorly understood due to the paucity of animal models, fully replicating the human disease. Previously, two quantitative trait loci (QTLs) associated with early cataract, AMD-like retinopathy, and some behavioral aberrations in senescence-accelerated OXYS rats were uncovered on chromosome 1 in a cross between OXYS and WAG rats. To confirm the findings, we generated interval-specific congenic strains, WAG/OXYS-1.1 and WAG/OXYS-1.2, carrying OXYS-derived loci of chromosome 1 in the WAG strain. Both congenic strains displayed early cataract and retinopathy but differed clinically from OXYS rats. Here we applied a high-throughput RNA sequencing (RNA-Seq) strategy to facilitate nomination of the candidate genes and functional pathways that may be responsible for these differences and can contribute to the development of the senescence-accelerated phenotype of OXYS rats.

**Results:**

First, the size and map position of QTL-derived congenic segments were determined by comparative analysis of coding single-nucleotide polymorphisms (SNPs), which were identified for OXYS, WAG, and congenic retinal RNAs after sequencing. The transferred locus was not what we expected in WAG/OXYS-1.1 rats. In rat retina, 15442 genes were expressed. Coherent sets of differentially expressed genes were identified when we compared RNA-Seq retinal profiles of 20-day-old WAG/OXYS-1.1, WAG/OXYS-1.2, and OXYS rats. The genes most different in the average expression level between the congenic strains included those generally associated with the Wnt, integrin, and TGF-β signaling pathways, widely involved in neurodegenerative processes. Several candidate genes (including *Arhgap33, Cebpg, Gtf3c1, Snurf, Tnfaip3*, *Yme1l1*, *Cbs*, *Car9 *and *Fn1*) were found to be either polymorphic in the congenic loci or differentially expressed between the strains. These genes may contribute to the development of cataract and retinopathy.

**Conclusions:**

This study is the first RNA-Seq analysis of the rat retinal transcriptome generated with 40 mln sequencing read depth. The integration of QTL and transcriptomic analyses in our study forms the basis of future research into the relationship between the candidate genes within the congenic regions and specific changes in the retinal transcriptome as possible causal mechanisms that underlie age-associated disorders.

## Background

The last years have seen great progress in understanding the pathophysiology of complex age-related diseases such as cataract and age-related macular degeneration (AMD): two of the major leading causes of visual impairment and blindness of elderly people in industrial countries. The molecular pathways underlying their onset and progression have yet to be described. Early stages of these diseases cannot be studied in humans, and existing animal models have severe limitations in recapitulating the disease progression [1-3].

We have previously shown that OXYS rats spontaneously develop a phenotype similar to human aging-associated disorders including retinopathy and cataract, with clinical, morphological, and molecular features similar to human AMD and senile cataract [4-8]. As we reported recently, neurodegenerative processes observed in OXYS rats are similar to those seen in Alzheimer's disease [9-11]. This animal model is successfully used to study the pathways and molecular alterations implicated in the development and progression of these disorders as well as to test new therapeutic interventions [11-16]. Nevertheless, the genetic architecture of this senescence-accelerated phenotype of OXYS rats remains poorly understood.

Analysis of quantitative trait loci (QTLs) is an unbiased genetic approach to studies of susceptibility genes and molecular pathways involved in diseases of complex etiology with a strong genetic component. The usual paradigm is to produce segregating populations derived from "affected" and "control" strains and to search for linkage of a complex trait to genetic markers using sophisticated statistical techniques. The methods of QTL analysis have long been used successfully in the studies of hypertension [17-19], neurodegeneration [20,21] and modifiers of pathological ocular phenotypes including retinal degeneration in mouse models [22-25]. On the other hand, some difficulties arise with the identification of QTL alleles at the level of causative genes [26]. Despite being confirmed and refined in location to a small interval spanning several megabases, a single QTL still harbors a number of genes (or does not generally contain annotated genes) and can act through a confluence of multiple interactions and molecular mechanisms.

Further testing for expression differences is one of the approaches aimed at identifying the genes underlying phenotypic differences [27]. In systems biology, identification of the underlying molecular pathways can be facilitated by high-throughput analyses, particularly at the proteomic and transcriptomic levels. RNA-Seq analysis, based on the next-generation sequencing technology, provides a far more precise quantification of transcripts than do other methods and allows for detection of novel transcriptomic features, e.g., novel exons and alternative splicing variants. The RNA-Seq technology has successfully been used for exploration of complex traits and, particularly, for identifying the genes related to retinal development and diseases [28-32].

Our previous QTL studies were focused on chromosome 1 and led to identification of two QTLs associated with early cataract, retinopathy similar to human AMD, and some behavioral aberrations in senescence-accelerated OXYS rats in a cross between OXYS and WAG rats. One QTL (named QTL1) was mapped to the medial region (100.6-188.0 Mbp) and the other QTL (named QTL2) - to the distal region (188.0-250.4 Mbp) of chromosome 1 [33]. Control WAG rats have no appreciable signs of cataract and retinopathy (unpublished data). Two interval-specific congenic strains, carrying OXYS-derived loci of chromosome 1 on the WAG strain, were generated to confirm our findings: WAG/OXYS-1.1 and WAG/OXYS-1.2. Both congenic strains develop early cataract and retinopathy but are clinically different from OXYS rats [33]. According to the results of a preliminary histological study (unpublished), the degenerative changes in the retina of the congenic rats occur in the context of mass migration of mononuclear phagocytes, e.g., into the ganglionic layer. This finding can serve as evidence of the development of an inflammatory process in the retinas of the congenic rats that is typical for patients with AMD, but not for OXYS rats.

The purpose of the present study was to identify candidate genes likely to be involved in the development of early cataract and retinopathy in rats of congenic and OXYS strains by analyzing the RNA-Seq data obtained from retinal RNA samples.

## Results

A previous study [33,34] showed that early cataract and AMD-like retinopathy in senescence-accelerated OXYS rats are associated with two QTLs located on chromosome 1, with the cumulative genomic length of 149.8 Mbp. We generated two interval-specific congenic strains, WAG/OXYS-1.1 and WAG/OXYS-1.2, carrying OXYS-derived chromosome 1 loci in the WAG genetic background. To map QTLs to the congenic loci and to facilitate nomination of the candidate genes responsible for the OXYS phenotype, we examined the retinal transcriptome of 20-day-old WAG/OXYS-1.1, WAG/OXYS-1.2, and OXYS strains using RNA-Seq. One sample of the WAG strain (three 3-month-old animals pooled) was processed, sequenced, and the reads were aligned to the rat genome (Rnor_5.0 assembly) to obtain a characteristic set of single-nucleotide polymorphisms (SNPs) for the second parental strain.

### Identifying the congenic regions on chromosome 1

**Figure 1:**
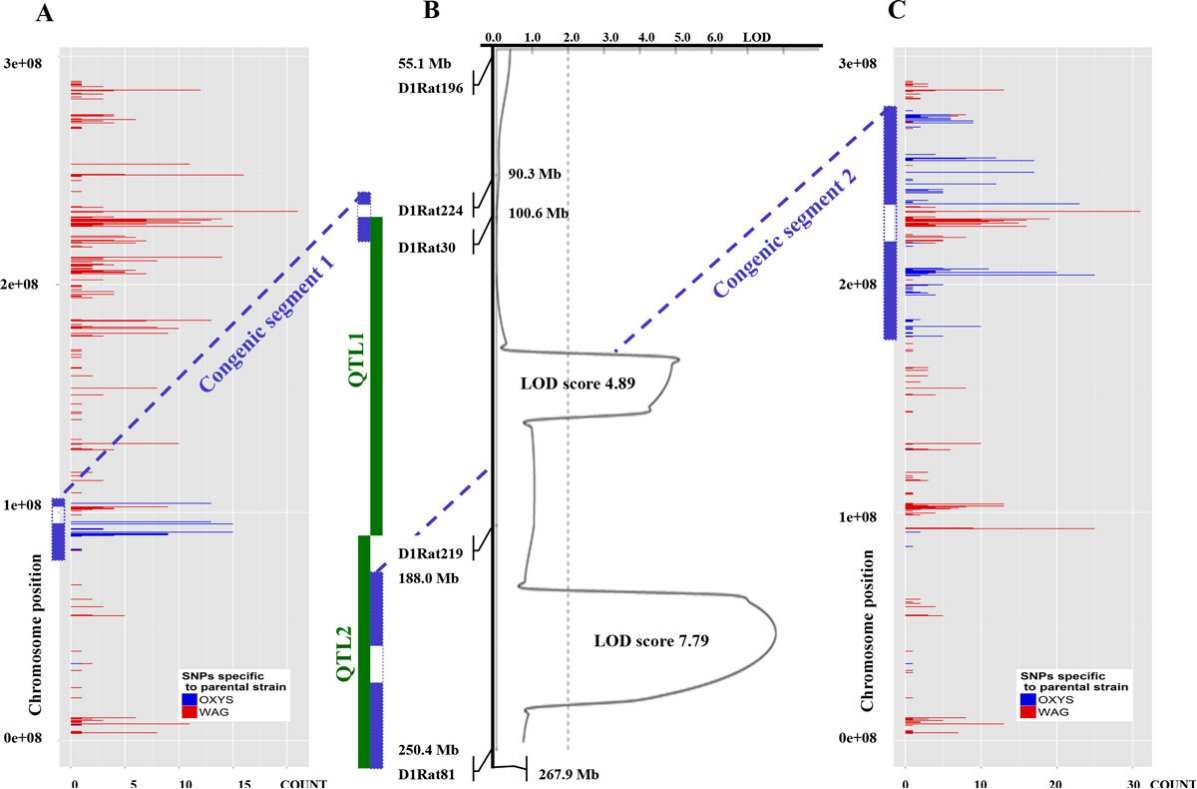
**The distribution of SNPs specific to the parental strains, on chromosome 1 of the congenic rats: WAG/OXYS-1.1 (panel A) and WAG/OXYS-1.2 (panel C)**. Panel B shows a plot of distribution of retinopathy linkage probability (LOD score) among the F2 progeny derived from OXYS and WAG strains at age 3 months (using microsatellite markers on chromosome 1). The dotted vertical line at LOD = 2.0 is the threshold for suggestive statistical significance. Red bars correspond to individual SNPs, specific to WAG rats, blue - to OXYS rats. COUNT - a number of coding SNPs for 1 Mbp. The OXYS-derived loci of chromosome 1 transferred to the recipient WAG genome during the construction of the corresponding congenic strains are shown as green (expected) and blue (the congenic segment) inserts, to the left of the linkage map. The blue dotted lines are designed to compare the certain congenic loci between the panels. Note, that the transferred locus was not what we expected in WAG/OXYS-1.1 rats.

The comparative analysis of SNPs specific to the OXYS, WAG, and congenic strains allowed us to identify the regions of chromosome 1 that came from the OXYS strain and were introgressed into the WAG/OXYS-1.1 and WAG/OXYS-1.2 constructed strains (Figure 1).

**Figure 2:**
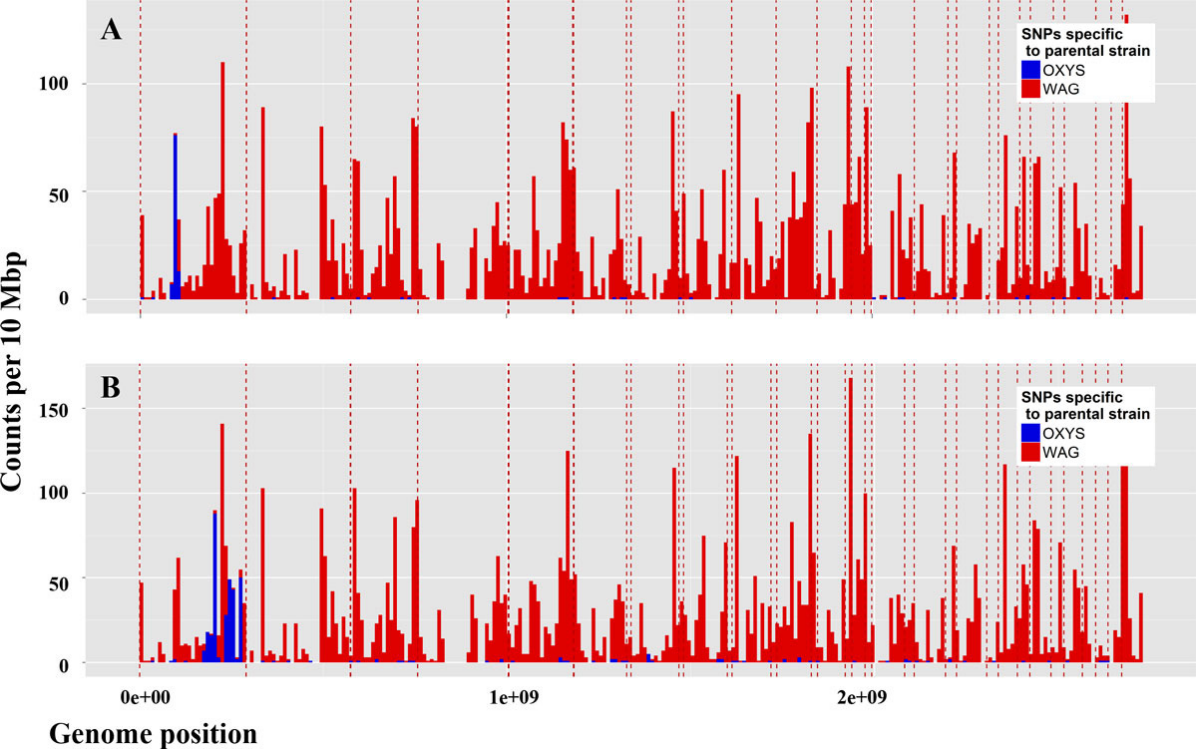
**The distribution of SNPs, specific to the parental OXYS and WAG strains, on all chromosomes of the congenic rats: WAG/OXYS-1.1 (panel A) and WAG/OXYS-1.2 (panel B)**. The vertical axis shows counts-the number of coding SNPs for 10 Mbp; the horizontal axis shows the length (in Mbp) of all chromosomes in genome, arranged in numerical order, where the chromosome boundaries are indicated by the red vertical dotted lines. The loci derived from OXYS parental strain are located on chromosome 1 of the congenic strains. Note, that no additional transferred extended loci are found on other chromosomes in each case.

We found that the OXYS segments introgressed into the WAG/OXYS-1.1 strain corresponded approximately to the positions of 8.9 × 10^7^-9.7 × 10^7 ^bp plus 1.04 × 10^8^-1.05 × 10^8 ^bp of chromosome 1. The OXYS segment introgressed into the WAG/OXYS-1.2 strain corresponded approximately to the positions of 1.78 × 10^8^-2.1 × 10^8 ^bp plus 2.34 × 10^8^-2.75 × 10^8 ^bp of chromosome 1. We found no additional transferred extended loci on other chromosomes in each case (Figure 2). So, the QTL-derived congenic segments were confined to the cumulative genomic segment of approximately 81.1 Mbp. There were 195 and 1013 ENSEMBL IDs within the WAG/OXYS-1.1 and WAG/OXYS-1.2 congenic segments, respectively, according to the results of data mining with the Encode project [35,36].

### The global transcriptome

**Figure 3:**
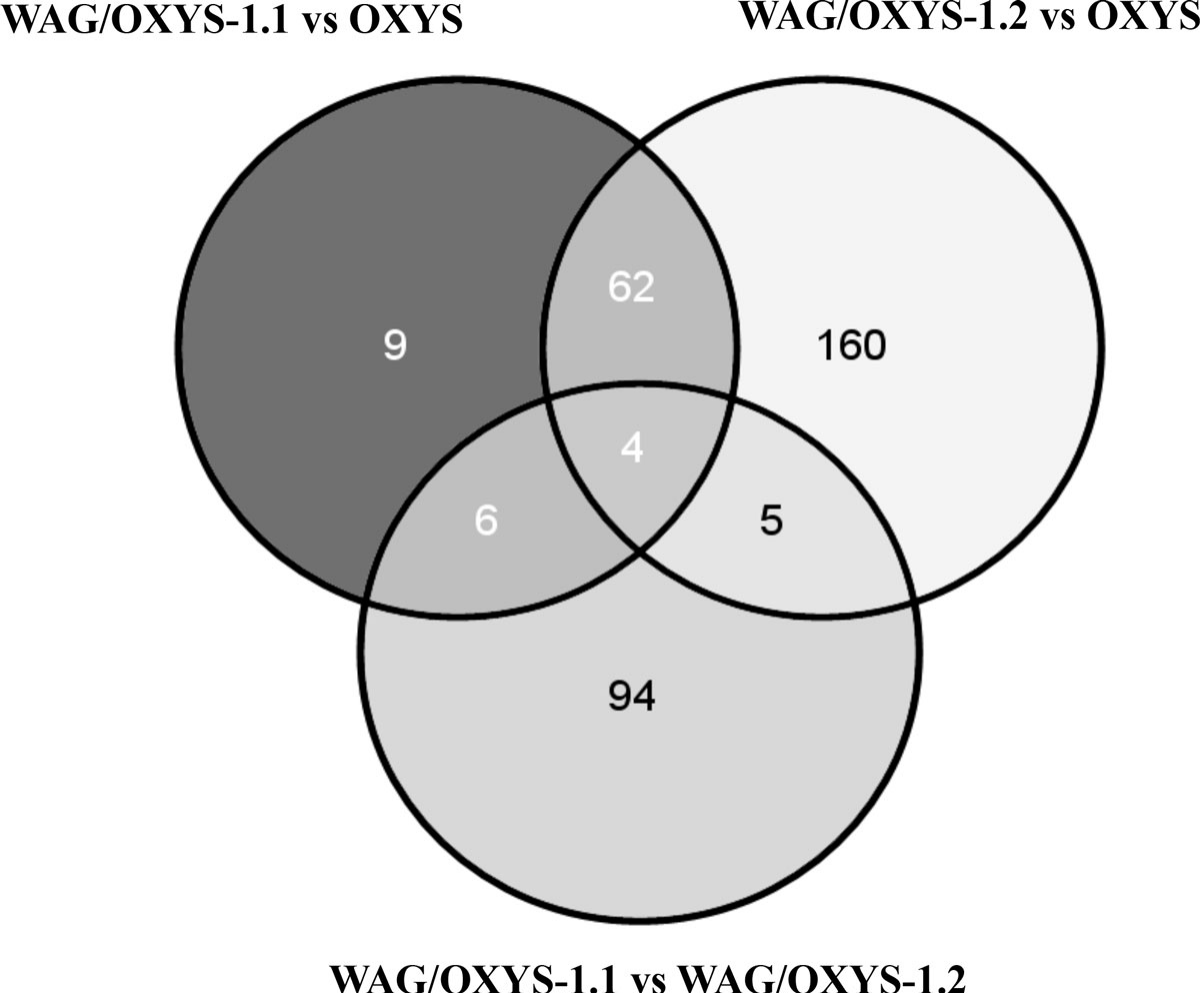
**The Venn diagram visualizing the overlapping results between the sets of ENSEMBL IDs for differentially expressed (DE) genes found in the WAG/OXYS-1.1 versus OXYS, WAG/OXYS-1.2 versus OXYS, and in WAG/OXYS-1.1 versus WAG/OXYS-1.2 (speculative observations) comparisons**. DESeq used only uniquely mapped to the reference genome reads at the cutoff of |log2FC |≥1.0; padj<0.1 for the WAG/OXYS-1.1 versus OXYS and WAG/OXYS-1.2 versus OXYS DE genes. In the WAG/OXYS-1.1 versus WAG/OXYS-1.2 comparison, the genes most different in the average expression level (pvalue < 0.05; |log2FC |≥1.0) were tested. The number of genes in each group is shown in the corresponding field.

Of 26405 genes in the reference genome rno5, our reads were uniquely mapped to 15442 genes (Additional file 1), with at least 10 counts on average. Differential expression of genes among the congenic WAG/OXYS-1.1 and WAG/OXYS-1.2 and parental OXYS strains was assessed using the DESeq package [37], resulting in the lists of 81 and 231 differentially expressed (DE) genes identified in the WAG/OXYS-1.1 versus OXYS and WAG/OXYS-1.2 versus OXYS comparisons, respectively (at padj<0.1 and the cutoff of |log2FC|≥1). We identified only 3 ENSEMBL IDs for genes significantly differentially expressed between the WAG/OXYS-1.1 and WAG/OXYS-1.2 congenic strains (padj<0.1): ENSRNOG00000009373, ENSRNOG00000042565, and ENSRNOG00000001273. Thus, in the WAG/OXYS-1.1 versus WAG/OXYS-1.2 comparison the strategy was to use Gene Ontology (GO) enrichment tools to test for functional categories that are enriched with the genes that are most different in the average expression level. Figure 3 shows the Venn diagram visualizing the overlapping results between the sets of DAVID IDs for DE genes found in the WAG/OXYS-1.1 versus OXYS, WAG/OXYS-1.2 versus OXYS, and in WAG/OXYS-1.1 versus WAG/OXYS-1.2 (speculative observations) comparisons. The DE genes were subjected to various further analyses.

### Pathway analysis

To identify pathways and biological functions associated (more than by chance) with the DE genes, we carried out gene annotation enrichment analysis using 2 online bioinformatic tools: DAVID [38] and WebGestalt [39,40]. The most representative GO terms are shown in the corresponding list of Additional file 2. A complete set of DAVID analyses of the entire lists of DE genes is presented in Additional file 2. Pathway analysis was also performed for separate up- and down-regulated DE groups (Additional file 3).

In the WAG/OXYS-1.1 versus OXYS comparison, DAVID revealed significant enrichment in GO terms relevant to the Srb-mediator complex, peroxisomes, and regulation of neurotransmitter release. Significant enrichment in GO terms, including the MHC class I protein complex, antigen processing and presentation, wound healing, a response to wounding, negative regulation of natural killer cell-mediated cytotoxicity, and Ca^2+^binding was obtained in other than the WAG/OXYS-1.2 versus OXYS DE gene set (Additional file 2). Some GO terms obtained using these two DE gene sets were relevant. Aside from GO terms related to extracellular matrix components, the analysis of both WAG/OXYS-1.1 versus OXYS and WAG/OXYS-1.2 versus OXYS DE sets of genes revealed categories related to fatty acid metabolic processes, the inflammatory response, and DNA ligation during DNA repair.

DAVID revealed enrichment in GO terms relevant to neuronal differentiation, blood vessel development, melanin biosynthetic processes, cell-cell signaling, membrane rafts, wide pore channel activity, regulation of transmission of nerve impulses, and transcription factor activity in a set of genes most variable in expression between the WAG/OXYS-1.1 and WAG/OXYS-1.2 congenic strains. The enriched KEGG pathway categories unique for this comparison were the Wnt signaling pathway, TGF-β signaling pathway, cadherin signaling pathway, melanogenesis, and Alzheimer's disease-presenilin pathway, where some named categories shared common genes from the five: *Bace2*, *Wnt16*, *Wnt6*, *Cdh1*, and *Cdh3*, members of the set of notably variable in average expression genes (Additional file 2, 3).

### Cross-referencing of QTL expression differences

The overlap between a gene's nomination via its presence within a QTL-derived congenic segment and its differential expression was analyzed first by cross-referencing of the lists of QTL genes with 81 and 231 DE genes identified in the WAG/OXYS-1.1 versus OXYS and WAG/OXYS-1.2 versus OXYS comparisons, respectively (Additional file 1). Thereby we identified 4 DE genes located only within the second congenic locus (introgressed into the WAG/OXYS-1.2 strain): *Zp2*, *Ifit1*, *Col17a1*, and *Snurf*.

### Polymorphisms

An allelic effect could be brought about at a different level of gene expression and as a result of an altered coding sequence. Further, the comparative analysis of non-synonymous coding SNPs (revealed for OXYS and congenic strains but not for WAG strain) was conducted with the focus only on the genes located within the congenic loci. Analysis using the online Variant Effect Predictor tool [41] revealed that non-synonymous substitution of amino acid residues in the protein products of 6 genes located within the congenic loci in WAG/OXYS-1.1 rats and of 13 genes located within the congenic loci in WAG/OXYS-1.2 rats may affect the function of the proteins (Table 1). The possible impact of an amino acid substitution on the structure and function of the protein was predicted using the SIFT algorithm, scale-invariant feature transform [42]. These 19 new candidate genes were not among the genes differentially expressed between the congenic carrier and OXYS strains (Additional file 1).

**Table 1:** Genes (within the congenic loci) containing non-synonymous coding SNPs with potential relevance to the senescence-accelerated phenotype in OXYS rats.

Gene symbol	Official gene name	Allele	CDS position	Protein position	AA change	Codon change	SIFT prediction (score)	Co-located variation
**Congenic locus in WAG/OXYS-1.1 strain**								

*Hps5*	Hermansky-Pudlak syndrome 5	C	667	223	R/G	Aga/Gga	tolerated (0.46)	-

*Hps5*	Hermansky-Pudlak syndrome 5	T	188	63	R/K	aGg/aAg	tolerated (0.25)	-

*Cic*	capicua transcriptional repressor	A	1517	506	C/Y	tGc/tAc	tolerated (0.08)	-

*Arhgap33*	RhoGTPase activating protein 33	T	3466	1156	D/N	Gat/Aat	deleterious (0)	-

*Lin37*	lin-37 homolog (*Caenorhabditis elegans*)	A	547	183	P/S	Cca/Tca	tolerated (0.18)	-

*Cebpg*	CCAAT/enhancer binding protein (C/EBP), γ	T	410	137	S/N	aGc/aAc	tolerated (0.45)	-

*Nudt19*	nudix (nucleoside diphosphate linked moiety X)-type motif 19	C	1015	339	S/G	Agc/Ggc	tolerated (0.42)	-

**Congenic locus in WAG/OXYS-1.2 strain**								

*Ccp110*	centriolar coiled coil protein 110 kDa	A	868	290	D/N	Gat/Aat	tolerated (0.17)	rs197588212

*Gtf3c1*	general transcription factor IIIC, polypeptide 1, α	A	6379	2127	R/C	Cgt/Tgt	-	rs8156681

*Gtf3c1*	general transcription factor IIIC, polypeptide 1, α	C	5692	1898	T/A	Aca/Gca	tolerated (0.75)	rs198509438

*Gtf3c1*	general transcription factor IIIC, polypeptide 1, α	C	5021	1674	N/S	aAt/aGt	tolerated (0.74)	rs106585674

*Rabep2*	rabaptin, RAB GTPase binding effector protein 2	C	1007	336	I/T	aTc/aCc	tolerated (1)	rs8153744

*Aldoa*	aldolase A, fructose-bisphosphate	C	493	165	M/V	Atg/Gtg	tolerated (0.58)	rs8160964

*Inpp5f*	inositol polyphosphate-5-phosphatase F	C	3075	1025	L/F	ttG/ttC	tolerated (0.44)	-

*Psat1*	phosphoserine aminotransferase 1	T	316	106	A/T	Gct/Act	tolerated (0.61)	-

*Tjp2*	tight junction protein 2	T	767	256	R/H	cGc/cAc	tolerated (0.59)	rs198995028

*Ak3*	adenylate kinase 3	T	368	123	L/Q	cTa/cAa	tolerated (0.66)	rs197195051

*Ric1*	RAB6A GEF complex partner 1	G	229	77	N/D	Aac/Gac	tolerated (1)	rs197794754

*RGD1311595*	similar to KIAA2026 protein	C	2613	871	I/M	atA/atG	tolerated (1)	rs198237726

*Sfxn3*	sideroflexin 3	A	927	309	N/K	aaC/aaA	tolerated (0.92)	rs8163692

*Nolc1*	nucleolar and coiled-body phosphoprotein 1	A	421	141	E/K	Gag/Aag	tolerated (0.61)	rs8165446

*Pdcd11*	programmed cell death 11	C	3005	1002	I/T	aTc/aCc	tolerated (1)	-

The SNP quality integral parameter threshold (in Samtools/Bcftools) was Qual> 180, and the consequence according to the Variant Effect Predictor tool, VEP [41] was "missense". Only 1 SNP, located in the *Arhgap33 *gene, was predicted to significantly affect protein structure (marked as "deleterious" by VEP). Position = position in base pairs (for CDS sequence) or in amino acid residues (for protein); AA = amino acid residue; colocated variation is presented according to the ENCODE data.

## Discussion

To identify candidate genes that are potentially involved in the development of early cataract and retinopathy in OXYS and derived congenic rats, we integrated transcriptomic data (obtained from retinas of 2 parental inbred: senescence-accelerated OXYS and WAG strains; and from retinas of 2 derivative congenic strains) with the results of previous QTL mapping of cataract and AMD-like retinopathy on chromosome 1 [33,34]. The analyses converged on a set of genes, including transcription factors (Table 1, Additional file 1), as the catching candidates (located within any of QTL-derived congenic regions or representing DE candidates), likely to be associated with the senescence-accelerated phenotype in rats.

### Congenic loci

In our previous study [33] the identification of genetic loci responsible for the trait of senescence-accelerated phenotype in OXYS rats was performed by QTL-analysis on hybrid F2 population aged 3-4 months and bred by a reciprocal cross between OXYS and WAG rats. Chromosome 1 showed significant associations between retinopathy and two loci: a) the locus marked by microsatellite markers D1Rat30 - D1Rat219 (named QTL1); and b) the locus marked by microsatellite markers D1Rat219 - D1Rat81 (named QTL2) (see Figure 1, panel B). Early cataract development was found significally associated with the locus in the region of the microsatellite markers D1Rat219 - D1Rat81 (QTL2). To determine the effects of these two QTLs, we generated two interval-specific congenic strains by transferring chromosome 1 regions from OXYS on WAG background with the transfer controlled by limiting microsatellite markers. Both congenic strains (named WAG/OXYS-1.1 and WAG/OXYS-1.2, respectively) display early cataract and retinopathy development. Thus, we confirmed that genes located in the analyzed regions of chromosome 1 are associated with the development of eye diseases in OXYS rats.

In the present study we determined the size and map position of QTL-derived congenic segments by comparative analysis of coding single-nucleotide polymorphisms (SNPs), which were identified for OXYS, WAG, and congenic retinal RNAs by the means of RNA-Seq. We found that a certain congenic locus, introgressed into the WAG/OXYS-1.1 strain, unexpectedly was not the locus in the vicinity of microsatellite markers D1Rat30 and D1Rat219 (Figure 1, panel B), but rather the locus marked by D1Rat224 - D1Rat30, which yielded LOD of 1.38 for linkage with cataract development in OXYS rats according to our unpublished data. Figuring out the reason for this discrepancy is beyond the objectives of the present study because WAG/OXYS-1.1 rats carry the OXYS-derived locus of chromosome 1 and develop cataract and retinopathy. Thus, the influence of genes located in the transferred locus on the disease development in OXYS and congenic WAG/OXYS-1.1 rats can be considered confirmed [43]. A possible explanation is that the congenic strain resulting from selecting only 1 locus of interest will have flanking donor chromosomal segments equal on average to 100/*N *cM on each side [43,44], where *N *is the number of backcrosses and 1 cM is equivalent to 2 × 10^6 ^bp of DNA in mice (and presumably in rats). After 8 backcrosses during the production of congenic strains in our study [33], the flanking DNA is on average ~12.5 cM on each side of the selected marker or 25 cM in size.

The locus, transferred from the OXYS to WAG/OXYS-1.2 strain was the expected one when introgressing the locus marked by D1Rat30 and D1Rat219 microsatellites. On other chromosomes, we did not find any extended OXYS-derived loci that were additionally transferred to any congenic strain (see Figure 2).

Here we report that the QTL-derived segments introgressed from OXYS rats are confined to the cumulative genomic length of ~81.1 Mbp and distinctly differentiate the WAG/OXYS-1.1 strain from WAG/OXYS-1.2 strain.

### The transcriptome

Because it has been suggested that variation in gene expression is an important contributor to the genetic architecture of complex traits [27], unraveling the changes in transcriptomic signatures should provide further understanding of the susceptible genetic loci that are involved in the pathogenesis of age-associated disorders, including AMD [32,45,46]. Recent genetic discoveries demonstrate that the pathogenic pathways in AMD are related to those in Alzheimer's disease among other disorders [47,48], thereby supporting the notion that AMD is an ocular manifestation of systemic disease processes [49], with a role for inflammation and immune processes in AMD pathogenesis [46,50,51].

We reported previously that AMD-like retinopathy is accompanied by downregulation of immune response genes and by changes in mRNA levels of a number of genes related to mitochondrial function in the OXYS retina at ages 3 and 18 months [52]. Impairment of the appropriate inflammatory response in OXYS rats was also observed in other tissues in a model of collagen-induced arthritis (our unpublished data, manuscript in preparation). In addition, we previously reported reduced strength of a delayed hypersensitivity reaction and a decline of T-cell-mediated immunity in OXYS rats [53]. One possible cause of the observed immune imbalance can be attributed to the accelerated thymus involution in OXYS rats [13]. Accordingly, we reported that the development of AMD-like retinopathy in OXYS rats is associated with an imbalance in immune and inflammatory responses [52] and carefully hypothesized that the impairment of immune function in OXYS rats may be associated with systemic neurodegenerative processes. The results of the present study support this hypothesis. When it comes over the GO terms, the imbalance of immune and inflammatory responses is observed at age 20 days between OXYS and both derivative congenic strains. Thus, the GO terms related to negative regulation of leukocyte-mediated cytotoxicity and positive regulation of T-cell-mediated cytotoxicity are downregulated in the WAG/OXYS-1.2 retina compared to OXYS retina; however, the GO term related to MHC class I protein complex is upregulated in this comparison. As for genes from the "Antigen processing and presentation" GO category, these are among up- and down-regulated sets between WAG/OXYS-1.2 and OXYS rats (Additional file 3). The results of pathway analysis are also suggestive of differences in DNA and lipid metabolism between the congenic and OXYS rats.

### Candidate genes in the congenic regions

Integration of RNA-Seq data from congenic rats with the results of QTL mapping reduces the number of positional candidates from the genes located throughout two QTL regions [9,34] to 19 candidate genes within the defined congenic loci. These genes contain non-synonymous coding polymorphisms that were revealed in the cDNA sequence of OXYS and derived congenic rats, but were not detected in the cDNA sequence of WAG rats (Table 1). None of 19 genes were defined as differentially expressed between the congenic carrier and parental OXYS strain (Additional file 1). The identified polymorphisms likely have functional consequences according to web-enabled Variant Effect Predictor Tool, thus may contribute to the senescence-accelerated phenotype of OXYS rats and to the differences in cataract and retinopathy development between congenic and OXYS strains. The consequences for only one of the coding non-synonymous SNPs described here, located in the *Arhgap33 *gene within the WAG/OXYS-1.1 congenic locus, are expected to significantly affect protein structure (labeled "deleterious" by the Variant Effect Predictor tool). *Arhgap33 *codes the member of the Rho family of GTPases, which can trigger signal transduction events in the regulation of actin cytoskeleton [54]. Members of this family modulate endocytic trafficking and α-secretase cleavage of the amyloid precursor protein (APP) [55], including interaction with BACE1 [56].

The label for the other missense SNPs in the congenic loci is "tolerated". Nevertheless, both congenic loci contain positional candidate genes that are of interest because of their potential contribution to the disease-associated pathways.

*Nudt19*, nudix (nucleoside diphosphate-linked moiety X)-type motif 19, is a potential biomarker of early stages of Alzheimer's disease [57] and, along with multiple Nudix family hydrolases, may function in mRNA decapping and maintenance of mRNA stability [58]. The CCAAT/enhancer-binding proteins (C/EBPs) are basic leucine zipper transcription factors that perform important functions in regulation of cell growth and differentiation, the inflammatory response, and hematopoiesis [59-62]. Biological functions of the protein product of *Cebpg*, CCAAT/enhancer-binding protein (C/EBP) γ, are not well understood, but a senescence-suppressing activity of C/EBPγ, which requires its ability to heterodimerize with C/EBPβ, was reported recently [63]. CEBPG is described as a modulator of C/EBP activity [64,65], a component of transcriptional regulation of B-cell development [62], and as a transcription factor primarily responsible for regulating transcription of key antioxidant and DNA repair genes in normal bronchial epithelial cells [66]. The protein product of *Lin37*, the lin-37 DREAM MuvB core complex component, is required for regulation of chondrocyte proliferation [67]. Another positional candidate gene within the WAG/OXYS-1.1 congenic locus is *Cic*, an ortholog of a Drosophila gene that is predominantly expressed during mouse granule cell development [68]. This gene is implicated in c-erbB (*Egfr*) signaling and is involved in abnormal migration of retinal pigment epithelium (RPE) [69]. As demonstrated on *Timp3*^−/−^mice, inhibition of EGFR signaling most likely reduces pathological retinal neovascularization in a mouse model of oxygen-induced retinopathy [70]. The EGFR signaling pathway is considered a possible link between extracellular signaling and regeneration-associated gene expression in the injured retina [71].

In the WAG/OXYS-1.2 congenic locus, we identified 3 non-synonymous coding SNPs within the *Gtf3c1 *gene, which is the general transcription factor IIIC, polypeptide 1α, coding a subunit of the general transcription factor IIIC [72]. The latter may regulate rearrangement of the nuclear architecture, allowing for coordinated expression of activity-dependent neuronal genes [73].

In addition, 4 candidate genes without coding polymorphisms, identified in the present study, were located in the second congenic locus and showed differential expression (Additional file 1) between the WAG/OXYS-1.2 carrier and parental OXYS strain: *Zp2*, *Ifit1*, *Col17a1*, and *Snurf*. Note, that this fact suggests a diverse transcriptional regulation which occurs against different genetic background.

The ZP2_51-149 _sperm-binding domain is necessary for human and mouse gamete recognition and penetration through the zona pellucida, the extracellular glycocalyx that surrounds an oocyte. GO annotations related to this gene include "acrosin binding" and "coreceptor activity" [74]. The *Col17a1*gene encodes the α chain of type XVII collagen, a transmembrane protein that is a structural component of hemidesmosomes, whose mutations result in a blistering disorder non-Herlitz junctional epidermolysis bullosa (JEB-nH) [75]. *Ifit1 *encodes a protein containing tetratrico peptide repeats that was originally identified as a protein induced upon treatment with interferon; it belongs to the "cytokine signaling in the immune system" category according to REACTOME pathway database [76]. This protein may inhibit viral replication and translation initiation [77,78].

*Snurf*, the SNRPN (small nuclear ribonucleoprotein N) gene, was found strongly upregulated in WAG/OXYS-1.2 retina when compared to OXYS (log2FC = -3,01324). This is the best characterized of genes associated with Prader-Willi syndrome (PWS) and Angelman syndrome (AS): neurobehavioral disorders that are caused by a deficiency in imprinted-gene expression from paternal and maternal chromosome 15q11-q13, respectively. The data from human and mouse models point to SNURF's being a protein that is produced along with SmN from a bicistronic transcript (this phenomenon is rarely observed in eukaryotes), with each cistron potentially playing a role in the disease [79,80]. The highly conserved activator sequence in *Snrpn *intron 1 demonstrates developmental dynamic changes of DNA methylation and has a methylation-sensitive enhancer activity in rodents; thereby it may control tissue-specific expression of transcripts [81]. Note, that the ubiquitously expressed transcription factor 1, Sp1, interacts with the SNURF protein, according to the Biological General Repository for Interaction Datasets, BioGRID [82]. Sp1 is reported to play a crucial role at early stages of hematopoietic specification [83] and to be involved in photoreceptor-specific transcription either directly or through competition [84] as well as in the regulation of expression of the vascular endothelial growth factor receptor 3 (VEGFR-3) [85].

### Candidate DE genes

Recently, we showed that senescence-accelerated OXYS rats are a promising model for studies of the mechanisms of the neurodegenerative processes similar to those seen in Alzheimer's disease [9-11]. In these rats, behavioral alterations and learning and memory deficits develop by the age of 3 months, accompanied by mitochondrial structural abnormalities, such as formation of cristae-free regions inside mitochondria by the age of 4 months. Moreover, we established a relationship between the retinopathy development in OXYS rats with alterations in the Alzheimer's disease metabolic pathway observed in the retina at the ages of 3 and 18 months [9].

In the present study, we uncovered candidate genes that are already differentially expressed in the retina of congenic rats at the age of 20 days - when compared with each other and with OXYS rats--and are associated with the Alzheimer's disease pathway or with maintenance of mitochondrion structure and function. Some of these genes are mentioned below.

### The comparison between WAG/OXYS-1.1 and WAG/OXYS-1.2 strains

Inbred WAG rats used in our study are of normal phenotype. So this comparison was intended to give the candidate DE genes which are associated with the different OXYS-derived loci of chromosome 1 transferred to congenic strains and potentially drive early cataract and retinopathy onset and progression in congenic rats. Simultaneously, we hypothesized that the same DE candidate genes can contribute to the development of the senescence-accelerated phenotype of OXYS rats.

As mentioned above, we identified only 3 ENSEMBL IDs for genes significantly differentially expressed between the WAG/OXYS-1.1 and WAG/OXYS-1.2 congenic strains (padj<0.1): ENSRNOG00000009373, ENSRNOG00000042565, and ENSRNOG00000001273. The last is related to *Psmg3*, proteasome (prosome, macropain) assembly chaperone 3, which is 1 of 14 distinct gene products essential for biogenesis of mammalian 20S proteasomes [86]. 20S proteasome is a catalytic core of the 26S proteasome, a central enzyme in the degradation of ubiquitin-conjugated proteins. This protein-degradation system might be a potential therapeutic strategy for AD and other tauopathies. The hypothesis that failure of proteasomal and non-proteasomal proteolytic clearance mechanisms leads to tau accumulation and progressive neurofibrillary degeneration in AD has been supported in a number of papers [87-89].

However, we could make some speculative assumptions regarding the results of the present study, based on the set of genes that are most variable in expression between the WAG/OXYS-1.1 and WAG/OXYS-1.2 congenic strains (with the cutoff of pvalue < 0.05; |log2FC|≥1). The list of most variable in expression genes from this comparison includes *Bace2*, β-site APP-cleaving enzyme 2, a paralog of *Bace1 *(a promising drug target in AD). We can hypothesize that such a difference is due to the differences in molecular networks, underlying the two congenic loci against the WAG background. Although normal functions of both enzymes, *Bace1 *and *Bace2*, are still unclear [90], it has been shown that astrocytes possess β-secretase activity and produce Aβ because of the activity of BACE2 [91]. BACE2 variants have an effect on the age of onset of dementia in people with Down syndrome [92]. Among other genes most variable in expression in this comparison are *Wnt16 *and *Wnt6 *(upregulated in the WAG/OXYS-1.1 group), the members of wingless-type MMTV integration site family and of the Wnt signaling pathway. The Wnt proteins perform a key function during various stages of retinal development and disease, e.g., they mediate angiogenesis and choroidal neovascularization, a severe complication of AMD [93]. Wnt proteins have also been implicated in AD [94,95]. The other gene upregulated in the WAG/OXYS-1.1 strain is the *Dkk1*gene, the product of which influences eye development from a defined developmental time point on, and is essential for lens separation from the surface ectoderm [96]. The function of *Dkk1 *has not been analyzed in detail in terms of eye development, but most likely the resulting protein acts in a dose-dependent manner, as a competent antagonist of canonical Wnt signaling [97,98].

NFATC4 (a nuclear factor of activated T-cells, cytoplasmic, calcineurin-dependent 4) is a transcriptional factor involved in the control of the flow of genetic information and in modulation of diverse cellular activities [99]. *Nfatc4 *exhibits a higher-than-average expression level in the WAG/OXYS-1.1 retina compared to WAG/OXYS-1.2. Accumulating evidence has demonstrated that NFATc4 exerts a proapoptotic effect in multiple diseases, and might participate in retinal ganglion cell apoptosis [100]. The proteoglycan decorin, encoded by the *Dcn *gene from the group, may also play a role in the differentiation of retinal ganglion cells [101] and is involved in hypoxic retinal damage, significantly reduce in expression in oxygen-induced retinopathy [102,103].

### The comparisons of retinal transcriptome profiles of the congenic and OXYS strains

These comparisons were intended to give the DE genes associated with phenotypic differences between parental OXYS and derivative congenic strains. Candidates from this set are potentially involved in molecular pathways underlying cataract and retinopathy progression, as well as in the development of other features of the senescence-accelerated phenotype in OXYS rats.

### Candidate DE genes unique in the WAG/OXYS-1.1 versus OXYS comparison

*Ephx2*, soluble epoxide hydrolase - a key enzyme in the metabolism and termination of action of epoxyeicosatrienoic acids, is significantly downregulated in WAG/OXYS-1.1 rats when compared to OXYS. Transgenic expression of *Ephx2 *in the endothelium is known to result in impaired endothelium-dependent vasodilation in the cerebral circulation [104].

*Cabp1*, a Ca^2+^-binding protein (CaBP), and the calcium sensor protein caldendrin [105] are alternatively spliced variants of a subfamily of CaBPs with high homology to calmodulin. Caldendrin is abundantly expressed in neurons and is thought to perform an important function at different stages of synaptodendritic Ca^2+ ^signaling [106,107]. Thus, caldendrin immunoreactivity is displayed by subpopulations of most retinal cell classes, with the exception of glial cells [108]. In the mouse brain, CaBP1/caldendrin is localized both pre- and post-synaptically, where it may regulate Ca^2+ ^signaling and excitability in selected groups of excitatory and inhibitory neurons [109].

The product of *Elovl1 *gene ortholog, ELOVL fatty acid elongase 1, participates in the metabolism of very long-chain fatty acids (VLCFAs) and a variety of inherited diseases, such as ichthyosis, macular degeneration, myopathy, mental retardation, and demyelination, which are caused by mutations in the genes encoding VLCFA-metabolizing enzymes [110,111].

*Cd14*, encoding the CD14 molecule, is significantly downregulated in WAG/OXYS-1.1 rats compared to OXYS strain. It is shown that fibrillar amyloid β (Aβ) can directly interact with Toll-like receptor 2 (TLR2), TLR4, and CD14 to induce microglial Aβ phagocytosis at the initial stages and neuroinflammatory responses at the late stages of Alzheimer's disease [112]. In particular, AD is characterized by the formation of insoluble deposits of Aβ associated with a robust microglia-mediated inflammatory response within the parenchyma of the brain. The involvement of CD14and TLRs in microglial activation was also reported, and it was suggested, that CD14 is a critical regulator of the microglial inflammatory response that acts to modulate Aβ deposition [113,114].

Is notable, that *Mrps10*, MRPS10 mitochondrial ribosomal protein S10, from this set of DE genes (upregulated in WAG/OXYS-1.1 rats when compared to OXYS) shows a tendency to be involved in regulation of the lifespan according to reverse transcriptase quantitative PCR (RT-qPCR) analysis using 2 genetic backgrounds [115].

### Candidate DE genes unique in the WAG/OXYS-1.2 versus OXYS comparison

The plasma membrane in eukaryotic cells contains microdomains that are enriched in certain glycosphingolipids, gangliosides, and sterols (such as cholesterol) and form membrane/lipid rafts that play a role in several cellular functions or characteristics including polarity of endothelial and epithelial cells, cell migration, mechano transduction, lymphocyte activation, neuronal growth, and signaling in a variety of disease settings [116]. *Sdc1*, syndecan 1, downregulated in the WAG/OXYS-1.2 strain when compared to OXYS rats, is a highly conserved multifunctional receptor [117]. Syndecan 1 was previously shown to undergo raft-dependent endocytosis upon clustering, regulating atherogenic postprandial remnant lipoproteins and molecules implicated in AD, during uptake of the biologically and medically important ligands, such as HIV-1 [118].

*Tnfaip3*, tumor necrosis factor α-induced protein 3 from this group, restricts and terminates inflammatory responses through modulation of the ubiquitination status of central components in the NF-κB [119], IRF3 (interferon-regulatory factor 3), and apoptosis signaling cascades. *Tnfaip3 *expression is necessary for prevention of chronic inflammation and autoimmune pathology according to studies on mice with full or conditional gene deletion [120]. Although *Tnfaip3 *was ruled out as the candidate gene for the chromosome 10 QTL for age-related retinal degeneration in mice [23], this gene was shown to downregulate adhesion markers, chemokine production, and adventitial angiogenesis, all of which are required for macrophage trafficking to sites of vascular injury in rats [121]. Recent experimental evidence shows that *A20/TNFAIP3 *is a susceptibility locus for human inflammatory and autoimmune disorders as well as a crucial gatekeeper preserving tissue homeostasis [122].

A human homolog of *Yme1l1 *has a similarity to mitochondrial conserved ATP-dependent proteinases (termed AAA proteases) that are embedded in the mitochondrial inner membrane, with the catalytic domain facing the mitochondrial intermembrane space [123]. This protein is required for resistance to apoptosis, cristae morphogenesis, and cell proliferation [124]. Nonetheless, how YME1L regulates mammalian mitochondrial function is still not clear.

Hypoxia-inducible factors (HIFs) mediate tissue-specific CBS (cystathionine β-synthase) expression and may augment cerebral vasodilation as an adaptive response to chronic hypoxia [125]. Moreover, ischemia/hypoxia increases accumulation of the CBS proteins in mitochondria [126], being a part of a possible general mechanism of oxygen-sensitive regulation of mitochondrial functions by linking the oxygenation level to accumulation/degradation of mitochondrial heme proteins. The *Cbs *gene is upregulated in the OXYS retina when compared to WAG/OXYS-1.2 rats.

Carbonic anhydrase IX (CA IX)-deficient *(Car9*^−/−^*) *mice exhibit abnormal locomotor activity, poor performance on a memory test, and vacuolar degenerative changes in the brain; these alterations seem to be age-dependent [127]. Our data show that *Car9 *expression in OXYS rats is downregulated in comparison with WAG/OXYS-1.2 rats.

The *Elf3 *codes transcription factor which is highly expressed in the retinal pigment epithelium (RPE) and can regulate important ocular genes, such as TIMP3, in vitro [128]. Variants of *Tap2 *have been shown to influence the antigenicity of MHC class I molecules by altering the MHC class I ligandome and to lead to reduced negative selection of CD8SP cells in rats [129].

### Candidate DE genes common for the WAG/OXYS-1.1 versus OXYS and WAG/OXYS-1.2 versus OXYS comparisons

Mediator (MED) complex is a multiprotein assembly playing a key role in eukaryotic transcription. Alterations of MED function are implicated in human diseases, e.g., alternative splicing of any MED gene may contribute to neovascularization [130]. Although neovascularization characterizes the end-stage of "dry" and "wet" forms of AMD, *MED8 *was found upregulated and *MED20 *- downregulated in both congenic strains compared to OXYS rats at the age of 20 days.

Two DE genes from this group are associated with maintenance of mitochondrion function: *Ehhadh *and *Acadm. Ehhadh*, L-bifunctional enzyme, is indispensable for the production of medium-chain dicarboxylic acids, thus providing an explanation for the coordinated induction of mitochondrial and peroxisomal oxidative pathways during fasting [131]. A synonymous polymorphic variation of ACADM, acyl-CoA dehydrogenase, C-4 to C-12 straight chain (exon 11) affects splicing efficiency and may alter fatty acid oxidation [132].

### Candidate DE genes in the Alzheimer's disease network

The DE genes found in WAG/OXYS-1.1 versus OXYS and WAG/OXYS-1.2 versus OXYS comparisons were also compared with human genes and their first-order interacting partners of the Alzheimer's disease network annotated in the NetAge database, which is used for the study of aging, longevity and age-related diseases [133]. Among several genes, we have got a potentially relevant candidate: fibronectin, *Fn1*, in the overlap. This gene encodes a glycoprotein, a member of a common signaling network which comprises over 40% of all proteins with multiple interactions in the human interactome [134]. Fibronectin binds to a complement protein C1q [135,136], which is induced in the brain in response to a variety of neuronal injuries, including AD, and blocks fibrillar amyloid-β (fAβ) neurotoxicity [137]. C1q is also well-known risk factor for retinal diseases related to oxidative stress, inflammation, and the complement cascade, e.g. is associated with complement alternative pathway of RPE cell death in AMD [138,139].

Summarizing our results, the QTL-derived loci of chromosome 1 in both congenic strains contain strong polymorphic candidates. Moreover, promising DE candidates are associated with each OXYS-derived locus on WAG/OXYS-1.1 and WAG/OXYS-1.2 genetic background. It appears that the trait of accelerated senescence under study is truly polygenic, with more than one gene contributing to OXYS-derived loci in each congenic strain even when some of these genes turn out to be not causative. As both congenic strains displayed early cataract and retinopathy but differed clinically from OXYS rats, it can be argued that the senescence-accelerated phenotype in OXYS rats is associated not just with the congenic loci under study. Thorough validation studies are necessary to address this question.

In general, the studies of variation in transcriptome snapshots in various tissues under different influences in congenic and parental strains can be used to explore and test molecular networks underlying variation in pathological phenotypes. It is possible that the systemic factors, such as environmental [140-142], have an influence on cataract and retinopathy development and contribute to differential expression of genes between strains. For some complex diseases, viral, bacterial, or environmental causes may ultimately prove to be of greater importance than disease-associated genes [141,143-145]. So, the combination of genetic factors with known environmental determinants indicates the highly complex nature of disease, but at the same time also offers insights into risk prediction [146]. Finally, some of the genes described here as candidates might be involved in the characteristic features of the senescence-accelerated phenotype that are not manifested during eye disease progression (e.g., behavioral variation observed in standard tests). Some of candidate genes may become expressed with age during development of a pathological phenotype. Thorough validation studies are needed to address this question, yet we can speculate that secondary changes in gene expression patterns between congenic and parental OXYS strains are triggered in the network associated with QTL-causing genes. In the present study, genes encoding transcriptional regulators that are polymorphic or differentially expressed in association with the congenic loci are especially good candidates.

We also identified significant transcript differences with potential relevance to disease development in a number of candidate genes, but we have not found SNPs within the coding regions of these DE genes. It is likely that other factors, e.g., the presence of non-coding SNPs in elements that regulate gene expression (including promoter and enhancer regions and the sites of transcription factor's binding) contribute to the observed transcriptional differences. Their detection requires further integrative studies.

Identification of master regulators of biological processes, including disease susceptibility and mapping of downstream gene networks, remains a big challenge for systems biology. Therefore, the efforts of researchers are focused on integrating results from diverse experiments, especially the data generated using high-throughput methods: genome-wide association (GWA) studies, chromatin immunoprecipitation followed by sequencing (ChIP-Seq), and RNA-Seq [147-149]. These approaches provide more complex information on expression levels, differential splicing, allele-specific expression, RNA editing, and fusion transcripts resulting from chromosomal translocations and other mutations [150-153], and also are helpful to investigate protein-DNA interactions [154]. As for the rat model, transcriptomic changes across multiple organs or developmental stages are recently beginning to be reported and integrated into open-access databases [149,155,156]. To our knowledge, this study represents the first analysis of the rat retinal transcriptome generated with 40 mln sequencing read depth by RNA-Seq technology.

## Conclusion

In this study, we carried out a comprehensive analysis of RNA-Seq data obtained for retinas of rats from parental and derivative congenic strains differing in the development of cataract and retinopathy. Furthermore, the genetic background of the trait under study obtained in previously published QTL-analysis was determined more accurately, and we overlaid the transcriptomic data obtained for 20-day-old rat retinas on the results. Thus, we obtained an unbiased list of candidate genes that may affect disease-associated pathways. In summary, our research of the congenic strains may facilitate dissecting of the complex genetic architecture of the senescence-accelerated phenotype in OXYS rats, although there is a need for further characterization of the congenic rats. With an improved understanding of the underlying genetic susceptibility, researchers can hope to identify therapeutic targets to nip AMD in the bud and to prevent its progression and vision loss in humans.

## Materials and methods

### Animals

All animal procedures were in compliance with the Association for Research in Vision and Ophthalmology statement for the Use of Animals in Ophthalmic and Vision Research and the European Communities Council Directive 86/609/EES. All manipulations of rats were approved by the Institutional Review Board #9 of the Institute of Cytology and Genetics, the Siberian Branch of the Russian Academy of Sciences (SB RAS), according to The Guidelines for Manipulations of Experimental Animals. Twenty-day-old male senescence-accelerated OXYS (n = 3), age-matched male WAG/OXYS-1.2 (n = 3) and WAG/OXYS-1.1 (n = 3) congenic rats, and 3-month-old WAG rats (n = 3) were obtained from the Breeding Experimental Animal Laboratory of the Institute of Cytology and Genetics, SB RAS (Novosibirsk, Russia). The OXYS rat strain was derived from the Wistar strain at the Institute of Cytology and Genetics as described earlier [157] and registered in the Rat Genome Database [158]. At this point, we have the 105^th ^generation of OXYS rats who exhibit spontaneously developing cataract and accelerated senescence syndrome inherited in a linked manner. The congenic rats were originally created as described earlier [33,34]. At this point, we have the 13^th ^generation of the congenic rats who exhibit spontaneously developing cataract and retinopathy. At age 4 weeks, the pups were weaned, housed in groups of 5 animals per cage (57 × 36 × 20 cm), and kept under standard laboratory conditions (22°C ± 2°C, 60% relative humidity, and 12 h light/12 h dark cycle). The animals were provided with standard rodent feed (PK-120-1, Ltd, Laboratorsnab, Russia) and water *ad libitum*. All experimental procedures were in compliance with the European Communities Council Directive of 24 November 1986 (86/609/EEC). All efforts were made to minimize the number of animals used and their discomfort.

### Tissue preparation

All rats were euthanized by CO_2 _asphyxiation. After decapitation the chorioretinal complex was excised rapidly on ice, placed in RNALater reagent (cat. #AM7020, Ambion), incubated at 4°C overnight in accordance with the manufacturer's protocol, frozen, and stored at −70°C prior to further processing.

### Sampling

The chorioretinal complex samples from three 20-day-old OXYS and three 20-day-old WAG/OXYS-1.2 rats were collected, processed, and sequenced individually, in triplicate. For 20-day-old WAG/OXYS-1.1 rats and 3-month-old WAG rats, 3 chorioretinal complex samples from 3 animals per strain were pooled to reduce the cost of the experiment. The RNA-Seq data obtained for WAG strain were not used for comparative analysis of retinal transcriptional profiles, as WAG rats were of other age group.

### RNA isolation

Frozen rat tissues were lysed in the TRIzol Reagent (cat. #15596-018, Invitrogen), and total RNA was isolated according to the manufacturer's protocols. RNA quality and quantity were assessed using Agilent Bioanalyser (Agilent) and the resulting RNA concentrations were determined using a Nanodrop 8000 spectrophotometer. The samples were stored at −70°C until further processing.

### Illumina sequencing

More than 40 mln single-end reads 50 bp long were obtained for each sample of retinal RNAs, using Illumina non-stranded sequencing on an Illumina GA IIx instrument at the "Genoanalitika" Lab, Moscow [159] in accordance with standard Illumina protocols (mRNA-Seq Sample Prep Kit, cat. #1004816). Briefly, polyA-tailed mRNA was purified from total RNA using Sera-Mag Magnetic Oligo (dT) beads and then fragmented into small pieces using divalent cations and heating. Using reverse transcriptase and random primers, first- and second-strand cDNAs were synthesized. The cDNA was processed in an end repair reaction with T4 DNA polymerase and Klenow DNA polymerase in order to blunt the termini. An "A" base was then added to the 3′ end of the blunt phosphorylated DNA fragments, and an Illumina adaptor with a single T overhang at its 3′ end was then ligated to the end of the DNA fragment, for hybridization in a single-read flow cell. After that, a size range of cDNA templates was selected, and these fragments were amplified on a cluster station using Single-Read Cluster Generation Kit v2. Sequencing-by-synthesis (SBS) of 50-nucleotide length was performed using SBS v4 reagents on a Genome Analyzer IIx running the SCS2.8 software (Illumina, cat. #FC-940-4001).

### Mapping and SNP discovery

After barcode trimming, the sequencing data were assessed for quality using the FastQC software and were mapped to the *Rattus norvegicus *reference genome assembly RGSC 5.0 (Ensemble release 75) using Bowtie 2 or TopHat v2.0.4. The SNP positions within the aligned reads (compared to the reference genome) were identified using the pileup function in SAMtools (v. 0.1.17) utilities [160]. Using the various filter commands, we predicted SNPs for various positions using the minimal mapping quality (Qual) of 100. These parameters ensured high-quality reliable mapping of the reads, which is important for variant calling. Using custom-made Perl scripts, we converted the VCF files into MySQL tables. Further analysis was based on the comparison of SNPs specifically present in OXYS, WAG/OXYS-1.1, WAG/OXYS-1.2, and WAG rats using MySQL 5.0 query, Oracle Corporation [161]. The free programming language R was used for the final data processing and visualization [162].

### Prediction of the SNP phenotypes

The SNPs--present in the specific congenic locus of chromosome 1 in OXYS and congenic rats (WAG/OXYS-1.1 or WAG/OXYS-1.2) but absent in control WAG rats--were considered possibly associated with the senescence-accelerated phenotype. The effect of a variant (amino acid substitution) on protein function was predicted using the Variant Effect Predictor web tool [41]; the consequence type, SIFT score, and prediction were calculated for each variant. SIFT scores of 0-0.05 were classified as "deleterious" and 0.05-1 - as "tolerated."

### Gene expression analysis

Sequencing data were preprocessed with Cutadapt [163] tool in order to remove adapters and low-quality sequences. The resulting reads were mapped on Rnor_5.0 reference genome assembly using TopHat2 [164]. The data were then converted into gene count tables using ENSEMBL gene annotaton data. The resulting tables were subjected to the analysis of differential gene expression with DESeq package [37]. The Benjamini-Hochberg correction for multiple testing was applied to the resulting p-values, and the genes with adjusted p-value < 0.1 were selected as differentially expressed for further study. Genes that failed to converge to Generalized Linear Model (GLM) in DE analysis were excluded.

### Pathway analysis

A bioinformatic approach was used to determine the biological context of the large amounts of gene expression (RNA-Seq) data. Gene lists from comparative analyses showing significant differences in gene expression were submitted to the free Database for Annotation, Visualization, and Integrated Discovery, DAVID [38]. In addition, functional annotation was conducted using the WEB-based Gene SeT AnaLysis Toolkit, WebGestalt [39,40]. All rat genes were set as a background. The NetAge database [133] was surfed to compare the rat DE genes from this study with human orthologs and their partners from the annotated Alzheimer's disease network.

## Availability of supporting data

The data sets supporting the results of this article are included within the article and its additional files.

## List of abbreviations

Mln: million; Mb: megabase (10^6 ^base pairs); cM: Centimorgan; AMD: age-related macular degeneration; RPE: retinal pigment epithelium; QTL: quantitative trait locus; LOD, LOD score: log10 likelihood ratio, comparing single-QTL model to the "no QTL anywhere" model; DE: differential expression; DEGs, DE genes: differentially expressed genes; NGS: next generation sequencing; GO: gene ontology; KEGG: Kyoto Encyclopedia of Genes and Genomes; SNP: single-nucleotide polymorphism

## Competing interests

The authors declare that they have no competing interests.

## Authors' contributions

This study was designed by EEK and NGK.

EEK designed the RNA-Seq experiments, sampled animals, performed bioinformatic analysis (including querying and interpretation of comparative SNP analysis data), analyzed the data, prepared figures, tables, and additional files, and drafted the manuscript. NIE carried out bioinformatic analysis including sequence alignment and DE analysis, and helped to draft the manuscript. LOB designed and developed the custom-made Perl scripts for the analysis pipeline and performed bioinformatic analysis (including screening for SNPs and preparation of figures). NGK supervised the experiments and drafted the manuscript. All authors read and approved the final version of the manuscript. Researches were the grant holders during the experimental procedures and data collecting.

## Supplementary Material

Additional file 1 **Contains RNA-Seq results by DESeq package**. Click here for file

Additional file 2 **Contains DAVID results for DE genes, DESeq**. Click here for file

Additional file 3 **Contains DAVID and WebGestalt results for separate groups of down- and up-regulated DE genes, DESeq**. Click here for file

## Declarations

Publication of this article has been funded by the BGRS\SB-2014 Organizing Committee and the Russian Science Foundation (RSF).

This article has been published as part of *BMC Genomics *Volume 15 Supplement 12, 2014: Selected articles from the IX International Conference on the Bioinformatics of Genome Regulation and Structure\Systems Biology (BGRS\SB-2014): Genomics. The full contents of the supplement are available online at http://www.biomedcentral.com/bmcgenomics/supplements/15/S12.
